# Phytotoxic Activity of Sesquiterpene Lactones-Enriched Fractions from *Cynara cardunculus* L. Leaves on Pre-Emergent and Post-Emergent Weed Species and Putative Mode of Action

**DOI:** 10.3390/plants13192758

**Published:** 2024-10-01

**Authors:** Daniela Rosa, Carlos Rial, Teresa Brás, Rosa M. Varela, Francisco A. Macías, Maria F. Duarte

**Affiliations:** 1 Alentejo Biotechnology Center for Agriculture and Agro-Food (CEBAL)/Polytechnic Institute of Beja (IPBeja), 7801-908 Beja, Portugal; daniela.rosa@cebal.pt (D.R.); teresa.bras@cebal.pt (T.B.); fatima.duarte@cebal.pt (M.F.D.); 2 MED—Mediterranean Institute for Agriculture, Environment and Development & CHANGE—Global Change and Sustainability Institute, Alentejo Biotechnology Center for Agriculture and Agro-Food (CEBAL), 7801-908 Beja, Portugal; 3 Allelopathy Group, Department of Organic Chemistry, Facultad de Ciencias, Institute of Biomolecules (INBIO), University of Cadiz, C/ Avenida Repuública Saharaui, s/n, 11510 Puerto Real, Spain; carlos.rial@uca.es (C.R.); famacias@uca.es (F.A.M.)

**Keywords:** allelochemicals, sesquiterpene lactones, *Cynara cardunculus* L., phytotoxicity, weed control, *Portulaca oleracea* L., mode of action, biopesticide

## Abstract

Sesquiterpene lactones (SLs) are compounds that are highly produced in *Cynara cardunculus* leaves, known for their phytotoxic activity. This study aims to assess SL-enriched fractions’ (cynaropicrin, aguerin B, and grosheimin) phytotoxic potentials and putative modes of action, compared to an initial extract, using two approaches: first, against a panel of nine weed species in pre-emergence, and then on *Portulaca oleracea* L.’s post-emergency stage. The SL-enriched fractions demonstrated greater phytotoxic activity when compared with the *C. cardunculus* leaf initial extract. The SL-enriched fractions had higher activity at root growth inhibition over the panel tested, doubling the activity in five of them at 800 ppm. Regarding the post-emergence bioassay, the SL-enriched fractions had a higher influence on the plants’ growth inhibition (67% at 800 ppm). The SL-effects on the plants’ metabolisms were evidenced. The total chlorophyll content was reduced by 65% at 800 ppm. Oxidative stress induction was observed because of the enhancement in MDA levels at 800 ppm compared to control (52%) and the decrease in SOD-specific activity from 4.20 U/mg protein (400 ppm) to 1.74 U/mg protein (800 ppm). The phytotoxic effects of the SL-enriched fractions suggest that they could be used for a future bioherbicide development.

## 1. Introduction

Agricultural crops’ productivity can decrease by 45–95% just due to the presence of weeds [1,2]. Synthetic herbicide application is considered to be the most successful method for weed control, but its widespread usage in the past several decades has resulted in a number of issues regarding human health and the environment, as well as herbicide resistance development [3]. With increasing restrictions on the use of synthetic herbicides, there is a growing demand for new solutions that combine efficiency, environmental sustainability, and cost. An alternative to synthetic pesticides is the application of natural phytochemicals. Numerous secondary metabolites produced by plants possess well-described allelopathic properties [4]. There are several advantages to using phytochemicals as tools for sustainable weed management, including alternate modes of action with more precise targets and easy biodegradation [1,5]. As a result, over the last decade, a wide range of phytochemicals have been identified and studied for their phytotoxic activity, including sesquiterpene lactones (SL) [6]. SLs represent a diverse group of natural compounds, with notable phytotoxic activity against standard target species as well as agricultural weeds of concern [6,7,8,9]. Although the SLs’ mode of action is still unclear, there is evidence highlighting the plasmatic membrane disruption, inhibition of membrane transport capability by dehydroazulanin C [10], inhibition of acetolactate synthase by costunolide, parthenolide, and 1,10-epoxyparthenolide [11], as well microtubule alteration auxin and ethylene imbalances by farnesene [5].

*Cynara cardunculus* L., commonly known as cardoon, is a multipurpose plant with a wide range of applications [12]. Our research team previously identified cardoon leaves as a natural source of SLs with values of 95 g/kg DW [13]. Their economic value extends to various sectors, including agriculture, pharmaceuticals, and energy production [12]. Cynaropicrin, grosheimin, and aguerin B have been identified, as most phytotoxic SLs present in *Cynara cardunculus* L. leaf extract (CcLE), with activity verified through wheat coleoptile bioassays and against weed species [14,15]. Although SLs demonstrate their potential for use as herbicides, their extraction and purification are expensive, time-consuming, and are generally conducted using hazardous solvents. In our previous work, it was evidenced that the use of ethanolic ultrasound-assisted extraction [16] and further purification using membrane technology via ultrafiltration membrane technology in diafiltration mode, effectively obtained SL-enriched fractions, with a 98.8% increase in SL purity [17]. As a result of the purification step, the phytotoxic activity against *Portulaca oleracea* L. was greater than that of the initial extract, both at the root and shoot growth inhibition. This study also demonstrates the successful purification of SLs from CcLE with enhanced phytotoxic activity with no need for harmful solvents, addressing an easy scaling up process, highlighting their potential for use in the phytochemical sector for the development of SL-based bioherbicides [17].

Thus, the goal of the current work is to assess the phytotoxic activity of SL-enriched fractions produced through ultrafiltration membrane technology against a wide panel of nine problematic weed species in agriculture at the pre-emergence stage. In addition, the effect of SL-enriched fractions, compared with the initial extract, on *Portulaca oleracea*’s post-emergence stage was also investigated to examine the possible mode of action for the morphology and physiology of the plant and evaluate their potential applications as a natural-based herbicide.

## 2. Results and Discussion

### 2.1. Phytotoxic Activity against a Panel of Weed Species

**Figure 1:**
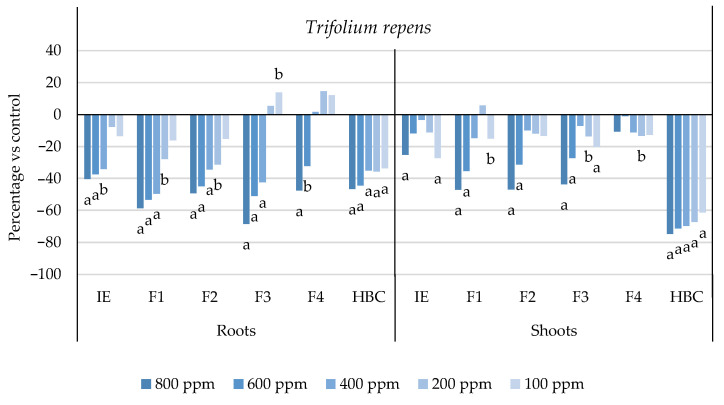
Effects of the initial extract (IE), SL-enriched fractions (F1–F4), and herbicide (HBC) on the growth of *Trifolium repens* roots and shoots. The values are expressed as the percentage difference from the control, and Welch’s test was used for statistical analysis. Letters *a* and *b* indicate significance for *p* < 0.01 and 0.01 < *p* < 0.05, respectively.

**Figure 2:**
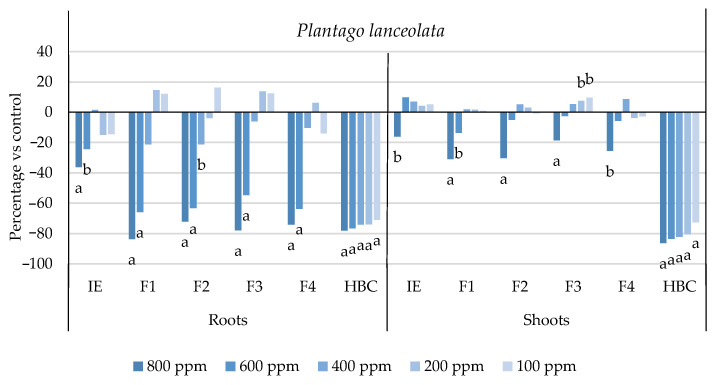
Effects of the initial extract (IE), SL-enriched fractions (F1–F4), and herbicide (HBC) on the growth of *Plantago lanceolata* roots and shoots. The values are expressed as the percentage difference from the control, and Welch’s test was used for statistical analysis. Letters *a* and *b* indicate significance for *p* < 0.01 and 0.01 < *p* < 0.05, respectively.

**Figure 3:**
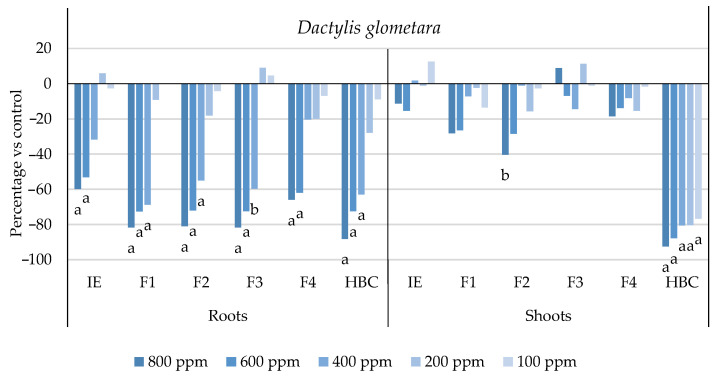
Effects of the initial extract (IE), SL-enriched fractions (F1–F4), and herbicide (HBC) on the growth of *Dactylis glomerata* roots and shoots. The values are expressed as the percentage difference from the control, and Welch’s test was used for statistical analysis. Letters *a* and *b* indicate significance for *p* < 0.01 and 0.01 < *p* < 0.05, respectively.

**Figure 4:**
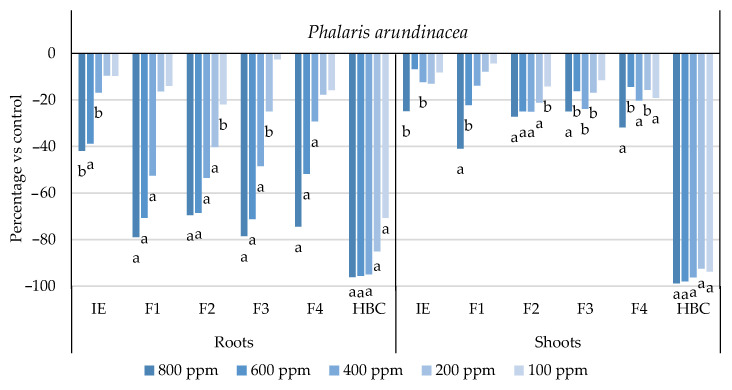
Effects of the initial extract (IE), SL-enriched fractions (F1–F4), and herbicide (HBC) on the growth of *Phalaris arundinacea* roots and shoots. The values are expressed as the percentage difference from the control, and Welch’s test was used for statistical analysis. Letters *a* and *b* indicate significance for *p* < 0.01 and 0.01 < *p* < 0.05, respectively.

**Figure 5:**
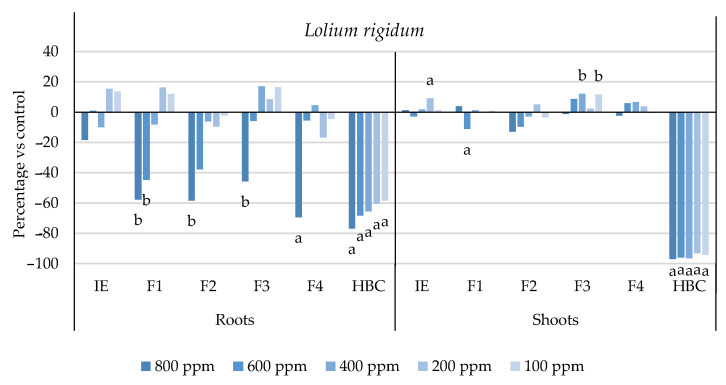
Effects of the initial extract (IE), SL-enriched fractions (F1–F4), and herbicide (HBC) on the growth of *Lolium rigidum* roots and shoots. The values are expressed as the percentage difference from the control, and Welch’s test was used for statistical analysis. Letters *a* and *b* indicate significance for *p* < 0.01 and 0.01 < *p* < 0.05, respectively.

**Figure 6:**
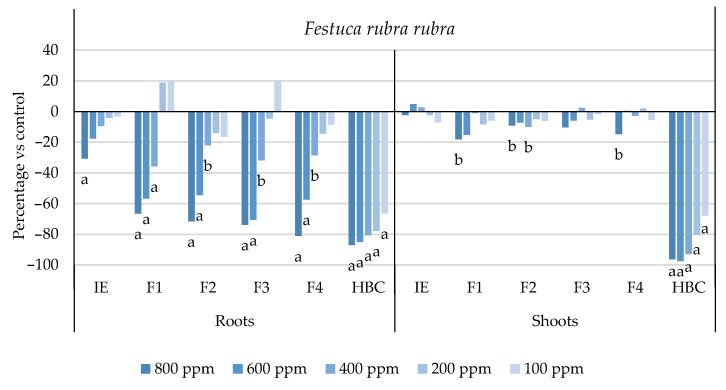
Effects of the initial extract (IE), SL-enriched fractions (F1–F4), and herbicide (HBC) on the growth of *Festuca rubra rubra* roots and shoots. The values are expressed as the percentage difference from the control, and Welch’s test was used for statistical analysis. Letters *a* and *b* indicate significance for *p* < 0.01 and 0.01 < *p* < 0.05, respectively.

**Figure 7:**
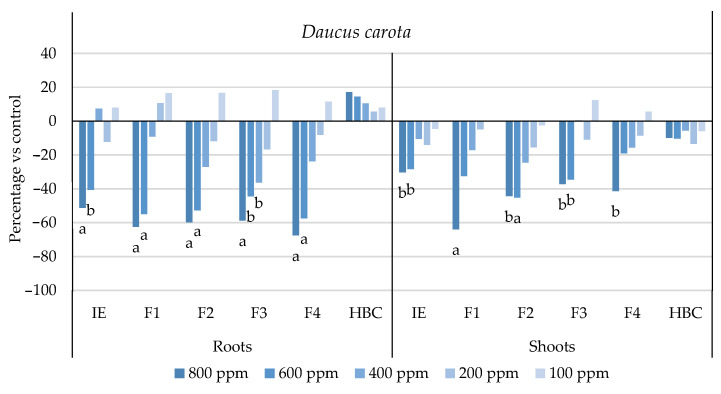
Effects of the initial extract (IE), SL-enriched fractions (F1–F4), and herbicide (HBC) on the growth of *Daucus carota* roots and shoots. The values are expressed as the percentage difference from the control, and Welch’s test was used for statistical analysis. Letters *a* and *b* indicate significance for *p* < 0.01 and 0.01 < *p* < 0.05, respectively.

**Figure 8:**
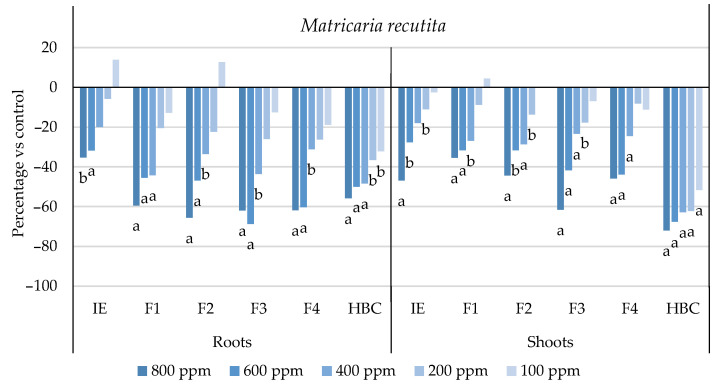
Effects of the initial extract (IE), SL-enriched fractions (F1–F4), and herbicide (HBC) on the growth of *Matricaria recutita* roots and shoots. The values are expressed as the percentage difference from the control, and Welch’s test was used for statistical analysis. Letters *a* and *b* indicate significance for *p* < 0.01 and 0.01 < *p* < 0.05, respectively.

**Figure 9:**
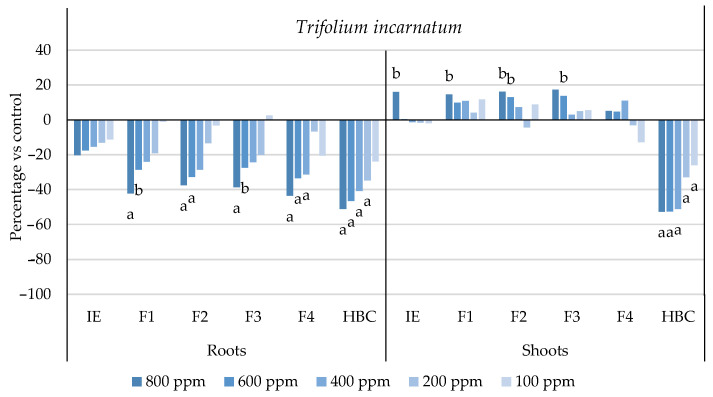
Effects of the initial extract (IE), SL-enriched fractions (F1–F4), and herbicide (HBC) on the growth of *Trifolium incarnatum* roots and shoots. The values are expressed as the percentage difference from the control, and Welch’s test was used for statistical analysis. Letters *a* and *b* indicate significance for *p* < 0.01 and 0.01 < *p* < 0.05, respectively.

The initial extract and SL-enriched fractions (F1–F4) were evaluated for their phytotoxicity against nine weed species: *Plantago lanceolata* L., *Phalaris arundinacea* L., *Trifolium repens* L., *Trifolium incarnatum* L., *Matricaria recutita* L., *Daucus carota* L., *Festuca rubra rubra* L., *Lolium rigidum* Gaudin, and *Dactylis glomerata* L. Table 1 shows that the enrichment of the fractions resulted in a 43% increase in cynaropicrin (from 198.89 to 348.11 ± 25.94 mg cyn/g of extract, on average, for the four fractions), a 45% increase in aguerin B (from 3.10 to 5.66 ± 0.49 mg agB/g of extract, on average, for the four fractions), and a 28% increase in grosheimin (from 5.49 to 7.57 ± 2.15 mg grosh/g of extract, on average, for the four fractions), as previously reported and discussed by Rosa et al. [17]. Thus, the observed phytotoxic activity is expected to be greater than the initial extract, as previously demonstrated against *Portulaca oleracea* L. [17]. Germination, root, and shoot length were evaluated with respect to the control, and the results are represented in Figure 1, Figure 2, Figure 3, Figure 4, Figure 5, Figure 6, Figure 7, Figure 8 and Figure 9.

**Figure 10:**
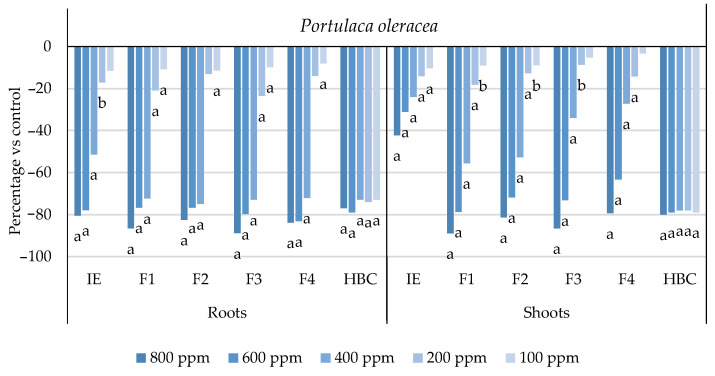
Effects of the initial extract (IE), SL-enriched fractions (F1–F4), and herbicide (HBC) on the growth of *Portulaca oleracea* roots and shoots. The values are expressed as the percentage difference from the control, and Welch’s test was used for statistical analysis. Letters *a* and *b* indicate significance for *p* < 0.01 and 0.01 < *p* < 0.05, respectively. (Adapted from [17]).

With respect to seed germination, neither the initial extract nor the SL-enriched fractions had a statistically significant negative effect on the weeds used (Supplementary Material File S1). With respect to root length growth inhibition, it was observed that SL-enriched fractions (F1–F4) exhibited higher phytotoxic activity compared with the initial extract, especially at *P. lanceolata* (Figure 2), which enhanced from 36% at 800 ppm to 84%, 72%, 78%, and 74% for F1, F2, F3 and F4, respectively, at 800 ppm; *P. arundinacea* (Figure 4) from 42% at 800 ppm to 79%, 70%, 78%, and 74% for F1, F2, F3, and F4, respectively, at 800 ppm; *F. rubra rubra* (Figure 6) from 31% at 800 ppm to 67%, 72%, 74%, and 81% for F1, F2, F3, and F4, respectively, at 800 ppm; and *M. recutita* (Figure 8) from 35% at 800 ppm to 59%, 66%, 62%, and 62% for F1, F2, F3, and F4, respectively, at 800 ppm. Furthermore, the SL-enriched fractions had phytotoxic activity against *L. rigidum* (Figure 5) and *T. incarnatum* (Figure 9), in contrast to the initial extract, which exhibited no phytotoxic activity. *P. oleracea* (Figure 10) was demonstrated to be highly susceptible to both extracts, with similar inhibitions levels between the initial extract and SL-enriched fractions at 800 and 600 ppm (values between 80 and 90%). However, at 400 ppm, root growth inhibition differs from 51% in the initial extract to values larger than 70% in the SL-enriched fractions. It is also significant to highlight the superior activity profiles exhibited by the SL-enriched fractions in comparison to the initial extract, demonstrating enhanced levels of inhibition on the root at concentrations lower than 800 ppm (Figure 1, Figure 2, Figure 3, Figure 4, Figure 5, Figure 6, Figure 7, Figure 8, Figure 9 and Figure 10). The enhancement of SL (cynaropicrin, aguerin B, and grosheimin) content in each fraction (Table 1) resulted in a greater phytotoxic activity when compared with the initial extract, especially at root level. Studies show that *C. cardunculus* leaf extract can effectively inhibit root length and have the least impact on germination upon different weed species [14,15,18]. According to Scavo et al. [14], ethanolic extracts inhibited root length more than 80% at 800 ppm over the weeds *Amaranthus retroflexus* L., *Portulaca oleracea*, *Stellaria media* L., *Anagallis arvensis* L., *Echinochloa crus-galli* L., and *Lolium perenne* L.

Regarding shoot growth inhibition, six of the ten weeds studied (*T. repens*, *P. lanceolata*, *P. arundinacea*, *D. carota*, *M. recutita*, and *P. oleracea*) were susceptible to initial extract and SL-enriched fractions, with different levels of inhibitions (Figure 1, Figure 2, Figure 4, Figure 7, Figure 8 and Figure 10, respectively). The difference between the initial extract and SL-enriched fractions was not evidenced for shoot inhibitions against *T. repens*, *P. lanceolata*, *P. arundinacea*, *D. carota*, and *M. recutita* (Figure 1, Figure 2, Figure 4, Figure 7 and Figure 8, respectively). The inhibition levels observed were not higher than 40%, except for *D. carota* (Figure 7), where F1 at 800 ppm inhibited 64%, and *M. recutita* (Figure 8), where F3 at 800 ppm inhibited 62%. *P. oleracea* (Figure 10) was the most affected weed on shoot growth inhibition by the SL-enriched fractions, where F1, F2, F3, and F4 inhibited more than 79% at 800 ppm and more than 63% at 600 ppm. *T. incarnatum* (Figure 9) was tolerant to both the initial extract and the SL-enriched fractions in terms of root and shoot growth inhibition. As stated by Rial et al. [15], the presence of cynaropicrin, aguerin, and grosheimin is corelated to the observed phytotoxic activity [15]. This study demonstrates that, following the chromatographic separation of the bioactive compounds found in the *C. cardunculus* leaf extract, the fractions containing higher SL concentrations exhibited phytotoxic activity comparable to the initial extract, suggesting that SLs are responsible for the extract’s bioactivity [15].

In regard to root inhibition, and because the various treatments had a greater effect on it, in a dose-dependent manner, the IC_50_ was calculated for each weed studied (Table 2).

According to the IC_50_ values obtained, an improvement in phytotoxic activity was clearly demonstrated based on the needed concentrations (initial extract vs. SL-enriched fractions) to inhibit 50% of root growth. According to the results, the decrease in IC_50_ values from the initial extract to SL-enriched fractions underly the phytotoxic activity of SLs, particularly the ones identified and quantified in the present study, namely cynaropicrin, aguerin B, and grosheimin (Table 1). Previous studies have demonstrated the role of parthenin, another SL, upon the germination and growth of *Ageratum conyzoides* L. [19].

Regarding the SL-enriched fractions’ phytotoxic activity against the weed panel, F1 had higher inhibitory effect against *P. lanceolata*, *D. glomerata*, and *L. rigidum*; F2 against *P. arundinacea*; F3 against *T. repens*, *F. rubra rubra*, *M. recutita*, and *P. oleracea*; and F4 against *D. carota*. According to the obtained results (Table 2) for each fraction in the various weed species, it is possible to conduct a phytotoxic activity analysis taking into account the IC_50_ value’s increasing order, which allows for the establishment of a ranking of activities into four levels, being the first level, the highest one, and the fourth lowest. According to this, it was found that F3 had the highest activity within the enriched fractions at the first level against four weed species, followed by F2 at the second level against three weed species.

To the best of our knowledge, a significant gap remains to be filled in studies related to the phytotoxic activity of *C. cardunculus* L. leaf extracts with respect to IC_50_ analysis. It appears that the enhancement of IC_50_ is predominantly associated with increased levels of SL cynaropicrin, aguerin B, and grosheimin within the respective fractions. In addition to the fundamental presence of these compounds, the interactions among them could be contributing to the differences in IC_50_ values among the fractions. According to the findings of Rial et al. [20], the binary mixtures formulated to assess synergistic effects among cynaropicrin, aguerin B, and grosheimin predominantly yielded additive and synergistic responses [20]. Nevertheless, the existence of an SL devoid of phytotoxic activity, specifically 11,13-dihydroxy-8-desoxygrosheimin, could potentially lead to additive, synergistic, or even antagonistic effects [17,20]. The fraction’s chemical composition in terms of SLs (Table 1) may clarify why F3 and F2 showed superior IC_50_ values in contrast to F4. The ratios of aguerin B and grosheimin were potentiated in F4 comparatively to F1, F2, and F3, as well as the permeated F4 fractions being equally rich in cynaropicrin.

**Figure 11:**
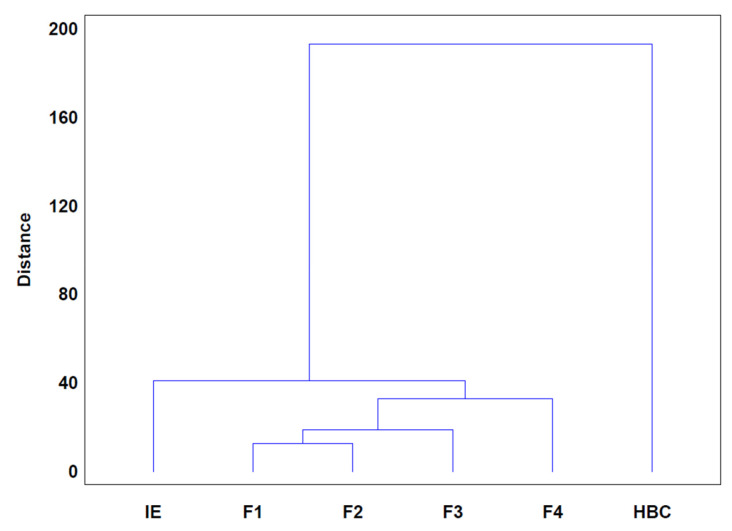
Cluster analysis of the phytotoxic effects of initial extract (IE), SL-enriched fractions (F1–F4), and the herbicide Stomp^®^Aqua (HBC) (positive control) on *Portulaca oleracea*, *Plantago lanceolata*, *Phalaris arundinacea*, *Trifolium repens*, *Trifolium incarnatum*, *Matricaria recutita*, *Daucus carota*, *Festuca rubra rubra*, *Lolium rigidum*, and *Dactylis glomerata* root and shoot growth inhibition.

A cluster analysis was performed using the root and shoot inhibition percentages at different concentrations of the initial extract, SL-enriched fractions, and commercial herbicide against the 10 weed panel (Figure 11).

The results show that the SL-enriched fractions were grouped, indicating similar levels of activity, F1 and F2 being more similar than F3 and, lastly, F4. Regarding the initial extract, our analysis shows that the phytotoxic behavior was different from the SL-enriched fractions placed in a different group. According to the phytotoxic activity described for the different weeds species, the results obtained from the present analysis are in accordance with a lower activity than SL-enriched fractions group. On the other hand, the commercial herbicide effect is significantly different from the other treatments, as noticed from their greater distance from the SL-enriched fractions group. The difference shown is associated with the accentuated activity among the panel tested. It should be noted that, despite demonstrating significant activity at the studied concentrations, at 800 ppm, the SL-enriched fractions exhibit a similar or even higher level of inhibition than the commercial herbicide, as seen with *P. oleracea* and *D. carota* at root and shoot levels, as well as *M. recutita*, *D. glomerata*, *T. repens*, and *P. lanceolate* at root levels.

**Figure 12:**
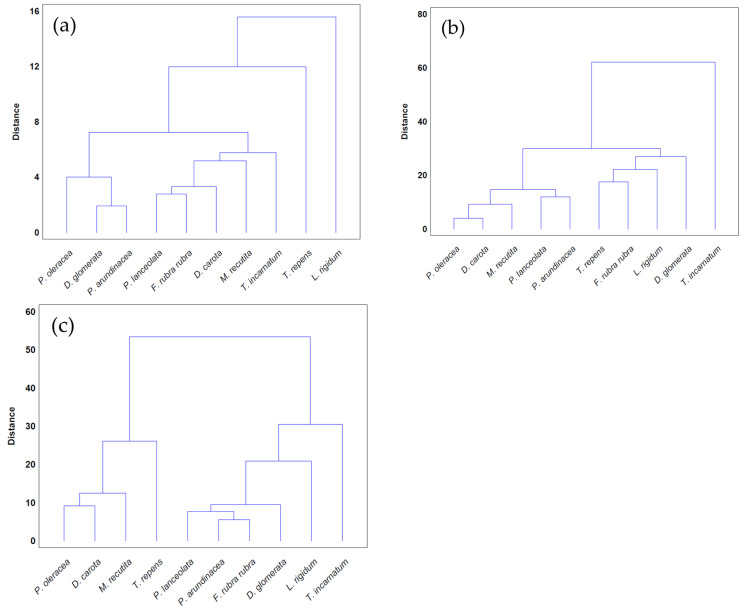
Cluster analysis of the susceptibility of weed species exposed to the SL-enriched fractions: (**a**) root growth; (**b**) shoot growth; and (**c**) root and shoot growth combination.

The cluster analysis regarding the susceptibility of the weed species studied, including *P. oleracea*, is represented in Figure 12.

The SL-enriched fractions (F1–F4) were evaluated in terms of root and shoot growth individually (Figure 12a,b), as well their combination upon the under-studied weeds panel (Figure 12c). In terms of root and shoot growth length inhibition, the phytotoxic effects were greater in the roots, as mentioned previously, according to a comparison of the distances obtained for the different weed species. In terms of root growth inhibition (Figure 12a), two major groups were formed on the basis of their susceptibility to SL-enriched fractions, with *P. oleracea*, *P. arundinacea*, and *D. glomerata* in one group, and *P. lanceolata*, *F. rubra rubra*, *D. carota*, *M. recutita*, and *T. incarnatum* in the other. *L. rigidum* and *T. repens* were the weeds that demonstrated higher differences being distanced from the others (Figure 12a). The groups formed might be explained by the phytotoxic activity observed in Figure 1, Figure 2, Figure 3, Figure 4, Figure 5, Figure 6, Figure 7, Figure 8, Figure 9 and Figure 10, where is possible to observe that the enhancement of SL content (Table 1) on the fractions had an inhibitory effect more accentuated on the first group comprising *D. glomerata* (Figure 3), *P. arundinacea* (Figure 4), and *P. oleracea* (Figure 10). For these weed species, the inhibitory effect at 800 ppm was around 80% and at 600 ppm was around 70%, statistically different from the control with 0.01< *p* < 0.05. As for the second group, comprising *P. lanceolata* (Figure 2), *F. rubra rubra* (Figure 6), *D. carota* (Figure 7), *M. recutita* (Figure 8), and *T. incarnatum* (Figure 9), it is possible to evidence that these weeds were less susceptible to the SL-enriched fractions since, at 800 ppm, the activity observed was between 70 and 80% of inhibition and between 600 ppm 40 and 65%, statistically different from the control with 0.01< *p* < 0.05 in most cases. *T. repens* (Figure 1) and *L. rigidum*’s (Figure 5) root length inhibition percentage observed was not higher than 65% at 800 ppm, meaning that the weeds were less susceptible to SL-enriched fraction root growth inhibition. With respect to shoot growth inhibition (Figure 12b), two primary groups were created: one with *P. oleracea* (Figure 10), *D. carota* (Figure 7), *M. recutita* (Figure 8), *P. lanceolata* (Figure 2), and *P. arundinacea* (Figure 4) (Group 1), and the other with *T. repens* (Figure 1), *F. rubra rubra* (Figure 6), *L. rigidum* (Figure 5), and *D. glomerata* (Figure 3) (Group 2). The two main groups formed might be explained by the higher susceptibility to the SL-enriched fraction in Group 1, with inhibitory percentages between 40 and 80% at 800 ppm, statistically different from the control with 0.01< *p* < 0.05 in most cases, and less susceptibility in Group 2, with maximum of inhibitory activity observed for *T. repens* (Figure 1) at 800 ppm of 47% (F1), statistically different from the control, and the other weeds with no more than 20% of inhibition. *T. incarnatum* (Figure 9) was the weed with the highest differences compared to the other weed species, related to the reduced susceptibility to SL-enriched fractions. When the effect of the different treatments on both root and shoot growth inhibition (Figure 12c) were combined, it was observed that two main groups were formed: one composed of four weeds, *P. oleracea* (Figure 10), *D. carota* (Figure 7), *M. recutita* (Figure 8), and *T. repens* (Figure 1), with similar susceptibilities to SL-enriched fractions, and a second group composed of the remaining six weeds.

Based on the results for phytotoxic activity in pre-emergent-stage weeds, *P. oleracea* was selected to assess the phytotoxic effect in adult plants mainly due to the high sensitivity of the roots to the initial extract and SL-enriched fraction, as indicated by IC_50_ values (Table 2). Therefore, a hydroponic bioassay was set up in order to evaluate the physiological and morphological effect upon adult *P. oleracea* plants through watering. In light of the findings from the cluster analysis, which showed that each SL-enriched fraction has similar phytotoxic activity for the majority of tested weeds, an equal mixture including F1–F4 was prepared for the current assays (Figure 11) and was evaluated in comparison to the initial extract.

### 2.2. Phytotoxicity Bioassay on Portulaca oleracea on Post-Emergence Stage

#### 2.2.1. Dry Weight vs. Control Ratio

**Figure 13:**
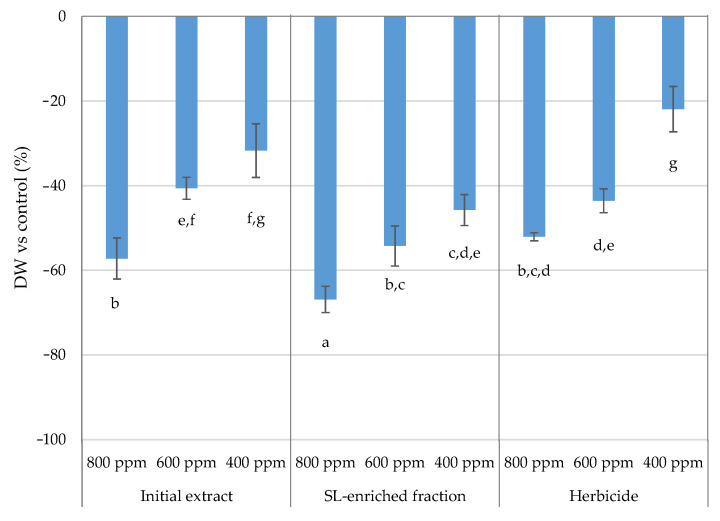
Values for *P. oleracea* DW determination at each treatment: IE—initial extract; SL-EF: SL-enriched fractions; HBC—herbicide (positive control). The values are expressed as percentage difference from control. Letters indicate significance between treatments and concentrations for *p* < 0.05, where a represents the higher negative value.

The effects of the initial extract and SL-enriched fractions upon *P. oleracea*’s growth development were measured through dry weight determination for each treatment (in relation to the control) (Figure 13).

Growth inhibition was dose-dependent, since growth inhibition increased from 400 ppm to 800 ppm when the plants were exposed to the treatments. The SL-enriched fractions’ treatment presented a greater profile of plant growth inhibition at the tested concentrations. At 800 ppm, there was greater growth inhibition (67%), followed by 54% and 46% at 600 ppm and 400 ppm, respectively. The effect of the initial extract was comparable to that of the herbicide treatment, but it was less active than the SL-enriched fractions (particularly at 800 ppm). The greater growth inhibition was indicative of the increased SL content in the fractions, with a higher effect observed in a dose-dependent manner. Prior research assessing the phytotoxic activity of naturally occurring phytotoxic compounds through hydroponic bioassays also noted that the various compounds inhibited the plants’ ability to grow and, as a result, reduced the plant’s dry weight at harvest. Based on the observed results, researchers hypothesized that the phytotoxic chemicals’ effects on root cell division, membrane permeability, and enzyme activity were the cause of the plants’ poor development and low biomass accumulation [21,22]. As in earlier research with SL parthenin, where a decline in the dry weight of *A. conyzoides* was noted as the concentration increased, the growth inhibition caused by SL-EF exposure may be related to this effect [19].

#### 2.2.2. Total Chlorophyll Content

**Figure 14:**
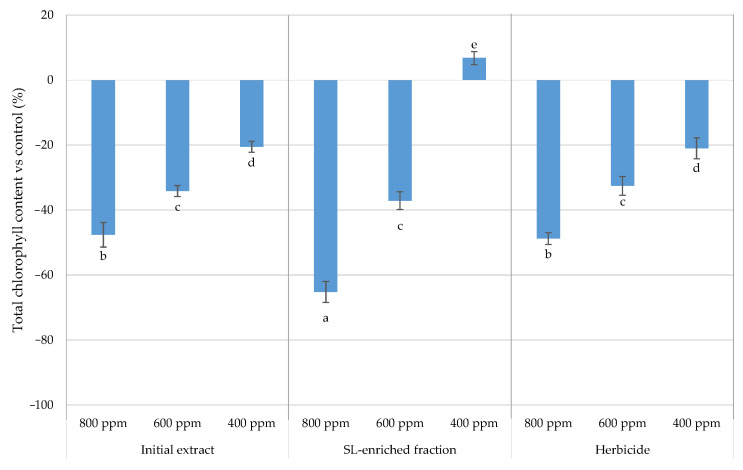
Total chlorophyll content in the leaf tissues of *P. oleracea* for each treatment: IE—initial extract; SL-EF: SL-enriched fractions; HBC—herbicide (positive control). The values are expressed as percentage difference from control. Letters indicate significance between treatments and concentrations for *p* < 0.05, where a represents the higher negative value.

The total chlorophyll content was determined for each treatment, and is presented with respect to the control (Figure 14).

A significant decrease in chlorophyll content was observed as the concentration increased from 400 to 800 ppm in the initial extract, SL-enriched fractions, and herbicide. At all concentrations tested, a similar effect was observed between the initial extract and the herbicide. SL-enriched fractions had a similar effect on total chlorophyl content than the initial extract and the herbicide at the concentration of 600 ppm. However, a greater decrease in the total chlorophyll content was observed at 800 ppm of SL-enriched extract, with the content decreasing by 65%.

A decrease in chlorophyll content reduces the plant’s photosynthetic efficiency, resulting in decreased energy generation and slower growth, which appear as observable symptoms such as chlorosis (yellowing of the leaves). The chlorophyll content in leaf tissues observed was dose-dependent and significantly lower at higher concentrations of SL-enriched fractions, where the SL content was considerably higher. These results are consistent with Motmainna et al. [23], with a decrease observed in total chlorophyll content in 22.78%, 45.31%, and 72.45% after *Ageratum conyzoides* L. exposure to 20 g/L, 40 g/L, and 60 g/L of *Parthenium hysterophorus* extract, respectively, compared to the control. These results were attributed to the allelopathic effect of the compounds present in the extract, such as the SL parthenin, since the reduction observed was dose-dependent [23]. Given the limited number of studies demonstrating the phytotoxic effect of SLs on chlorophyll content following exposure, some examples of this effect are provided to help interpret the results. Batish et al. [24] also reported the phytotoxic effect of lemon-scented eucalypt oil against *Cassia occidentalis* and *Echinochloa crus-galli* under field conditions. A drastic decrease in chlorophyll content was observed for both species at 8% of volatile oil. *C. ocidentalis* was more susceptible, showing a 85% decrease when exposed to 2.5% of volatile oil [24]. The authors of the mentioned study associated these findings with a possible alteration in leaf diffusibility, transpiration rate, and stomatal aperture, affecting photosynthesis [24]. Olive mills waste water and fig aqueous extract have been associated with decreases in chlorophyll content between 53% and 27% in lettuce plants, respectively. This reduction in chlorophyll levels may be a result of cell damage caused by the allelochemicals present [25]. Thymol, a natural phenolic monoterpene widely produced by different species, was applied through sub-irrigation at 300 µM against *Arabidopsis thaliana* for 16 days. This treatment led to a significant reduction in chlorophyll a (30%) and chlorophyll b (31%) in *Arabidopsis thaliana* plants, attributed to lower levels of carotenoids, which play an important role in protecting against photoinhibition [26]. The limited protection against the photodegradation of chlorophylls is directly related to the formation of reactive oxygen species (ROS), and thereby induces oxidative stress in the plant [26].

#### 2.2.3. SOD Activity Measurement

**Figure 15:**
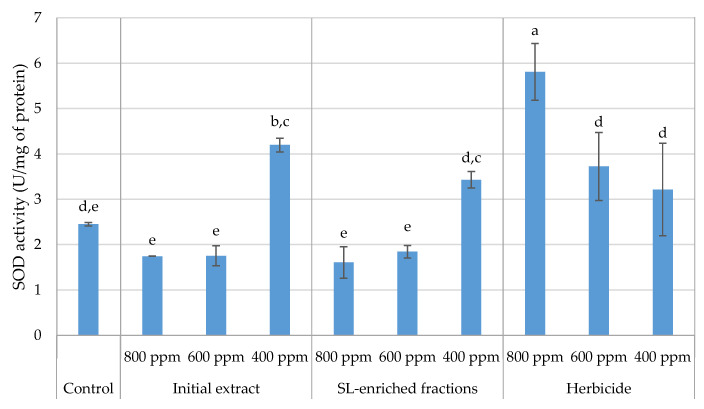
SOD activity in the leaf tissues of *P. oleracea* in response to different treatments IE—initial extract; SL-EF: SL-enriched fractions; HBC—herbicide (positive control). The values are expressed as U/mg of protein. Letters indicate significance between treatments and concentrations for *p* < 0.05, where a represents the higher value.

Plants respond to oxidative stress induced by xenobiotics through a complex interplay of antioxidant defense mechanisms, with superoxide dismutase (SOD) being a key player in this physiological response [27]. Therefore, SOD activity was assessed in the leaf tissue of *P. oleracea* treated with the initial extract, SL-enriched fractions, and the herbicide on hydroponic media (Figure 15).

SOD activity decreased as the concentration of both the initial extract and SL-enriched fractions increased from 400 to 800 ppm, indicating statistically similar behavior, with 1.74 and 1.61 U/mg of protein SOD activity, respectively, for 800 ppm and 1.75 and 1.84 U/mg, respectively, for 600 ppm. On the other hand, a different behavior was observed for the herbicide treatment where the SOD activity increased in response to increasing herbicide concentration, with a maximum activity of 5.81 U/mg protein.

In response to oxidative stress, plants upregulate the expression of SOD to counteract the accumulation of superoxide radicals and maintain cellular homeostasis [27]. The increase in SOD activity with increasing HBC concentration might be related to the upregulation of SOD expression. However, the extent of the SOD response and the overall antioxidant defense capacity can vary depending on the plant species, the type of xenobiotic, and the duration of exposure. In both the initial extract and the SL-enriched fractions, an increase in SOD activity was observed at 400 ppm and then a decrease was observed between 600 and 800 ppm, which might indicate that the antioxidant enzyme mechanism of defense was compromised at higher SL concentrations. To the best of our knowledge, this is the first report on the impact of SLs on SOD-specific activity; a few studies on this effect are provided to shed light on the effect that was found. A similar behavior was observed in Wang et al.’s study, where SOD activity inhibition was reported upon chlorimuron-ethyl herbicide treatment with different durations of exposure in wheat (*Triticum aestivum*) when compared to the control [28]. In another study, two synthetic herbicides, methyl viologen and benzyl viologen, belonging to the bipyridylium herbicide family, and the natural compound juglone, extracted from walnut trees, were used to induce oxidative stress in developing and germinated maize scutella. The reported results demonstrated that, at both plant stages, the juglone doses induced an enhancement of SOD activity up to 0.01 mM at the 28 days post-pollination stage and 1 mM at the 5 days post-imbibition stage, and then dropped at higher concentrations [29]. Peppermint water extract of allelochemical stress induction on radish (*Raphanus sativus* L.) growth and development was evaluated [30]. Regarding SOD activity in response to stress inductions, it remarkably decreased from 184.7 to 59.5 U mg^−1^ min^−1^ of fresh weight in a dose-dependent manner in the extract. In other words, higher concentrations of the extract with stronger phytotoxic properties decreased the efficacy of O^2−^ scavengers [30].

In light of the results obtained, and to substantiate the findings acquired, it would be advantageous to assess, in a subsequent study, the H_2_O_2_ conversion activity facilitated by the enzymes, catalase (CAT), ascorbate peroxidase (APX), or glutathione peroxidase (GPX), as well as the non-enzymatic components, ascorbic acid (AsA) and glutathione (GSH) [31]. The quantification of the aforementioned conversion could yield additional insights into the capacity of sesquiterpene lactones (SLs) to influence the antioxidant defense system at a specific juncture within the signaling cascade induced by oxidative stress. According to Hegab et al. [32], the bioactive compound tricin, extracted from jungle rice (*Echinochloa colona* L.), was implicated in the accumulation of H_2_O_2_, which, in turn, led to an increase in malondialdehyde (MDA) content [32]. These observations were correlated with a potential inhibition of SOD and peroxidase (POX) enzymes, resulting in the accumulation of H_2_O_2_ [32]. Associating those SLs derived from both the initial extract and SL-enriched fractions may inhibit SOD-specific activity, as demonstrated by the results; the additional measurement of other enzymatic and non-enzymatic activities could elucidate whether the observed enhancement of MDA content in the SL-enriched fraction at 800 ppm is a direct consequence of the inhibition of enzyme/non-enzyme systems responsible for converting H_2_O_2_.

#### 2.2.4. Determination of Lipid Peroxidation

**Figure 16:**
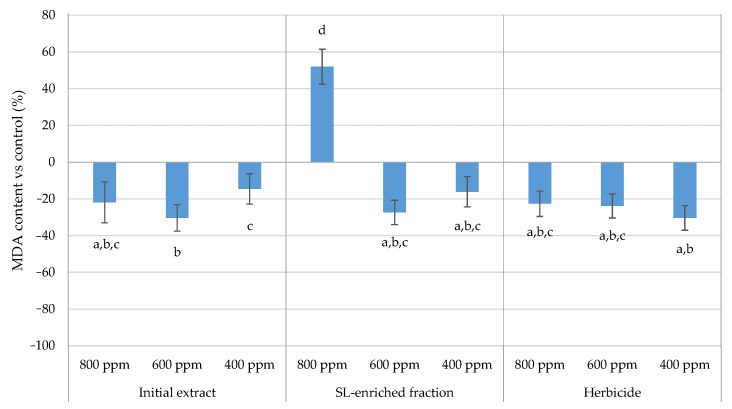
MDA content in the leaf tissues of *P. oleracea* for each treatment: IF—initial extract; EF: SL-enriched fractions; HBC—herbicide (positive control). Values are expressed as percentage difference from control. Letters indicate significance between treatments and concentrations for *p* < 0.05.

MDA assessment is commonly used as a biochemical marker to assess the extent of lipid peroxidation and oxidative stress in plants, since it is one of the end products of lipid peroxidation. The MDA content is represented in respect to control in Figure 16 for each treatment.

This study reveals that the induction of lipid peroxidation occurred only in the 800 ppm SL-enriched fraction, as demonstrated by a 52% increase in the MDA level in the leaf tissue compared with that in the control.

The plasma membranes of cells and organelles are composed primarily of lipids. As reactive oxygen species levels increase, normal cellular activities are disrupted, and oxidative stress is aggravated by the formation of lipid-derived radicals and lipid peroxidation [33]. The exposure to the SL-enriched fraction at 800 ppm resulted in lipid peroxidation induction during the 12 days of exposure. Investigations including the use of natural extracts have also shown an increase in MDA concentration in response to oxidative stress. Motmainna et al. reported that MDA content was significantly higher in *A. conyzoides* when exposed to 60 g/L of *P. hysterophorus* extract, with an enhancement of 152% compared to the control [23]. *P. hysterophorus*’ effect on total chlorophyll content might be related to the enhancement of MDA by inducing the degradation of carotenoids and the indirect degradation of chlorophyll and therefore inducing oxidative stress by accumulation of ROS. Given the limited number of studies demonstrating the phytotoxic effect of sesquiterpene lactones on lipid peroxidation induction following exposure, an example is provided to help understand the findings. In another study, it was also observed that MDA content enhanced on lettuce roots and leaves when exposed to the olive mill waste water aqueous extracts and leaf aqueous extracts of olive and fig between 80.4% and 52.4%, respectively. This elevation in MDA levels indicates higher lipid peroxidation and oxidative damage in the plant’s membranes [25].

## 3. Material and Methods

### 3.1. Reagents

The commercial herbicide Stomp^®^Aqua was provided by BASF Agricultural Solutions (Ludwigshafen, Germany). Hoagland and Arnon nutrient solution, xanthine, xanthine oxidase, nitro blue tetrazolium, vermiculite, polyvinylpyrrolidone, trichloroacetic acid, Tris-HCl, bovine serum albumin (BSA), and Bradford reagent were purchased from Sigma-Aldrich (St. Louis, MO, USA). Dimethyl sulfoxide was acquired from Panreac (Barcelona, Spain). Acetone was purchased from VWR (Radnor, PA, USA). River sand and clay were purchased from Mascotas Ávila (Seville, Spain).

### 3.2. Cynara cardunculus L. SL-Enriched Fractions Obtention

SL-enriched fractions were obtained from a previous work [17]. Briefly, *Cynara cardunculus* L. leaf extract was fractioned by using the ultrafiltration membrane Suez™GH with a molecular weight cut-off of 2000 Da in diaultrafiltration mode. The feed samples and cumulative and instantaneous permeated samples were collected every 24 h for 8 days, resulting in 8 permeate fractions. Experiments were carried out until a maximum loss of 10% of cynaropicrin was achieved. Fractions 1 to 4 were chosen for the present study, given the enhancement in SL concentration per gram of extract with respect to the initial extract concentration (Table 1).

### 3.3. Phytotoxic Activity Bioassay against a Panel of Weed Species

The phytotoxic activity of the initial feed and SL-enriched fractions obtained was evaluated against a panel of weed species that consisted of the following: *Plantago lanceolata* L., *Phalaris arundinacea* L., *Trifolium repens* L., *Trifolium incarnatum* L., *Matricaria recutita* L., *Daucus carota* L., *Festuca rubra rubra* L., *Lolium rigidum* Gaudin, and *Dactylis glomerata* L. (Cantueso Natural Seeds, Córdoba, Spain). Bioassays were conducted in Petri dishes as described by Rial et al. [15], with some modifications. Briefly, the initial extract (IE) and SL-enriched fractions (F1–F4) were tested at concentrations of 100, 200, 400, 600, and 800 ppm on 20 seeds of each species. The commercial herbicide Stomp^®^Aqua (HBC) was used as a positive control at the same concentrations (BASF Agricultural Solutions, Germany). Four replicates were prepared for each treatment. Seeds were further incubated at 25 °C in the dark for 8 days, except for *Dactylis glomerata*, which was incubated for 13 days. The germination rate and root and shoot lengths were measured using a Fitomed system, which allowed for automatic data acquisition and statistical analysis via associated software. The data were analyzed statistically using Welch’s test, with significance fixed at 0.01 and 0.05. The results are shown as a percentage difference from the control. Zero represents control, positive values correspond to stimulation, and negative values correspond to inhibition.

### 3.4. Calculation of IC_50_ Values and Cluster Analysis

IC_50_ values were determined by fitting activity data to a sigmoidal dose–response model with GraphPad Prism 5.00 software. The cluster analysis was performed using Statgraphics 19 software (Statgraphics Technologies, Inc., The Plains, VA, USA). Cluster analysis was used to group the initial extract, SL-enriched fractions, and the herbicide with similar phytotoxicity behaviors in order to evaluate the weed species’ susceptibilities to the initial extract and SL-enriched fractions [34]. The phytotoxic effect of *P. oleracea*, as described in an earlier study by Rosa et al. [17], was included in the cluster analysis.

### 3.5. Phytotoxicity Bioassay on the Metabolism of Adult Portulaca oleracea

The evaluation of the phytotoxic potential of *P. oleracea* metabolism was based on the hydroponic bioassay previously described by Anese et al., with some modifications [21]. Ten seeds of *P. oleracea* were grown in pots filled with washed river sand, vermiculite, and clay at a ratio of 1:1:1. After fifteen days of growth, plants were individually placed in 50 mL falcon flasks filled with approximately 56 g of glass beads (LAB Comercial Equipments, S.L., Barcelona, Spain) as a substrate. Hoagland and Arnon [35] nutrient solution at 1/3 ionic strength was used for acclimation of the plants. Fifteen plants were used per treatment, and fifteen milliliters of test solutions were added per falcon. The test solutions, initial extract (IE), SL-enriched fraction (SL-EF), and commercial herbicide Stomp^®^Aqua (HBC) were pre-dissolved in dimethyl sulfoxide (5 µL/mL solution) and diluted in nutrient solution to concentrations of 400, 600, and 800 ppm. Nutrient solution plus dimethyl sulfoxide was used as a control. Aluminum foil was used to protect the roots from light, and the top of the falcons was coated with parafilm in order to prevent the solution evaporating. The experiment was conducted over 12 days in a climatic greenhouse regulated at 25 °C with a photoperiod of 12 h. After the 12-day trial, the plants were harvested and the biomass’ dry weight, total chlorophyll content, superoxide dismutase-specific activity, and malondialdehyde content were measured.

#### 3.5.1. Measurement of Dry Weight

The entire plant of four *P. oleracea* was used for each treatment. They were dried with a heater at 70 °C for 72 h and weighed to estimate their dry weight (DW). After these data were obtained, the dry weight treatment/control (DW treatment/control) ratio was calculated for each treatment.

#### 3.5.2. Total Chlorophyll Content Determination

For the extraction of chlorophylls from *P. oleracea* leaves, 0.1 g of fresh leaves were homogenized with 10 mL of 80% acetone using a mortar. The homogenized solution was centrifuged at 10,000× *g* for 10 min, and the supernatant was collected. To the volume obtained, acetone was further added to a final volume of 10 mL. The procedure was performed in triplicates for each treatment and control. Absorbance was measured at wavelengths between 645 nm and 663 nm, and the total chlorophyll content was calculated according to Porra et al. [36]. The results were expressed in µg/g of fresh weight and treatment vs control percentage.

#### 3.5.3. Enzymatic Extract Preparation for Biochemical Parameter Determination

Fresh leaves of treated and control plants (1 g) were homogenized in a mortar using liquid nitrogen in an extraction buffer (20 mM Tris-HCl in 1% polyvinylpyrrolidone, pH 7.4) at 20% (*w*/*v*). The extracted solution was centrifuged at 10,000× *g* for 30 min at 4 °C to remove debris. The supernatant was collected and stored at −80 °C until further use.

#### 3.5.4. Superoxide Dismutase (SOD)-Specific Activity Measurement

The reaction solution was prepared in a 96-well plate by mixing 216.8 µL of phosphate buffer 50 mM (pH 7.5) with 5 mM of EDTA, 32 µL of nitro blue tetrazolium 1.0 mM, and 32 µL of xanthine 50 mM. After equilibration at 5 min, 3.2 µL of xanthine oxidase (1 U/mL) was added, and the absorbance was recorded at 560 nm during 5 min. A blank was also prepared where, instead of xanthine oxidase addition, it was replaced by deionized water. After 5 min, 16 µL of the enzymatic extract was added, and the decrease in observance was recorded up to 15 min. SOD activity was calculated according to the equations described by Roy et al. [37]. Total protein content was determined for SOD-specific activity determination according to the Bradford method [38], with some modifications. The reaction mixture was prepared in a 96-well plate composed of 5 µL of standard compound/enzymatic extract and 250 µL of Bradford reagent. The plate was incubated until a strong blue color was achieved, it did not exceed 60 min of incubation, and the absorbance measured 595 nm. Bovine serum albumin (BSA) was used as a standard compound at concentrations between 62.5 and 1000 µg/mL, obtained using two-fold dilution. Total protein content was expressed per gram of fresh weight and as a percentage of treatment vs. control.

#### 3.5.5. Malondialdehyde (MDA) Content Measurement

A lipid peroxidation-induced evaluation was conducted by measuring the malondialdehyde (MDA) content. The enzymatic extract was added to either 20% trichloroacetic acid (*w*/*v*) or 0.5% thiobarbituric acid (*w*/*v*) in 20% trichloroacetic acid (*w*/*v*). After 30 min of incubation at 95 °C, these combinations were cooled in an ice bath and centrifuged at 3000× *g* for 10 min. The supernatant was collected, and the absorbance was measured at 532, 600, and 440 nm. The MDA equivalents (nmol.mL^−1^) were calculated using the equations described by Hodges et al. [39]. The results are expressed per gram of fresh leaves and treatment vs. control percentage.

## 4. Conclusions

In this study, SL-enriched fractions (cynaropicrin, aguerin B, and grosheimin) had higher activity at root growth inhibition on the nine-weed panel tested, doubling the activity in five of them at 800 ppm. The effects of the SL-enriched fractions on the morphological and physiological parameters of *P. oleracea* L., compared with initial extract, were demonstrated. The observed decrease in biomass weight might be due to interferences with cell division and elongation, corroborated by impaired physiological functions [40]. The reduction in chlorophyll content, which alters the plant’s ability to perform photosynthesis effectively, consequently affects plant growth [41]. Additionally, the generation of reactive oxygen species leading to oxidative stress in plants is linked to exposure to phytotoxic compounds [41]. With increasing concentrations of SL-enriched fractions, there was a noted decrease in SOD-specific activity. This decline may be attributed to a compromised antioxidant enzyme defense system caused by elevated SL concentrations. As a result, a weakened antioxidant defense system initiates a cascade of events, potentially damaging cellular components such as lipids [41]. Lipid peroxidation was also observed in the presence of SL-EF at 800 ppm, indicated by the increased MDA content, a byproduct of the reaction. This process, induced by SL-triggered oxidative stress, might compromise membrane integrity and cellular functions. A subsequent study regarding the H_2_O_2_ conversion activity facilitated by the enzymes and non-enzyme constituents of the antioxidant defense system should be undertaken to clarify the impact of SLs on the oxidative induction response in plant systems.

In conclusion, the present study demonstrates a greater phytotoxic activity of SL-enriched fractions vs the initial extract for root growth inhibition of several weed species in their pre-emergence stage. SLs demonstrated their influence on the plant morphological and physiological parameters studied. SL-enriched fractions produced higher inhibition on plant growth by influencing chlorophyll content in a dose-dependent manner and by inducing oxidative stress, which was evidenced at 800 ppm of SL-EF. These results highlight the possible application of the SL-enriched fraction as a bioherbicide. Future work will focus on the experimental design of an ecological formulation and its field application for the management of sustainable agriculture.

## Figures and Tables

**Table 1:** SL content (mg of compound/g of extract) for initial feed (IE) and permeate fractions (F1–F4) obtained through Suez™GH membrane diafiltration (Suez, NJ, USA) (adapted from [17]).

Fractions	Cynaropicrin (mg/g of Extract)	Aguerin B (mg/g of Extract)	Grosheimin (mg/g of Extract)	Total SL Concentration (mg/g of Extract)
Initial extract (IE)	198.89 ± 2.03 ^c^	3.10 ± 0.02 ^d^	5.49 ± 0.02 ^c^	207.48 ± 2.07 ^c^
Fraction 1 (F1)	376.20 ± 3.86 ^a^	5.86 ± 0.09 ^b^	7.18 ± 0.17 ^b^	389.24 ± 4.12 ^a^
Fraction 2 (F2)	313.42 ± 8.46 ^b^	5.35 ± 0.05 ^c^	6.83 ± 0.68 ^b^	325.60 ± 9.19 ^b^
Fraction 3 (F3)	349.87 ± 6.82 ^a^	5.16 ± 0.10 ^c^	5.64 ± 0.13 ^c^	360.67 ± 7.05 ^a^
Fraction 4 (F4)	352.95 ± 19.88 ^a^	6.25 ± 0.08 ^a^	10.64 ± 0.29 ^a^	369.84 ± 20.25 ^a^

Letters indicate significance *p* < 0.05 between IE and F1–F4 for each SL and total SL concentration, where *a* represents the higher value (one-way ANOVA and Tukey’s multiple comparison test).

**Table 2:** IC_50_ values of root growth inhibition of the initial extract (IE), SL-enriched fractions (F1–F4), and commercial herbicide (HBC) determined for each weed species tested.

	IC_50_ (ppm)					
	IE	F1	F2	F3	F4	HBC
*T. repens*	>800 ^a^	663.6 ± 1.2 ^b^	>800 ^a^	527.9 ± 1.1 ^c^	>800 ^a^	>800 ^a^
*P. lanceolata*	>800 ^a^	524.0 ± 1.1 ^e^	549.6 ± 1.1 ^d^	588.6 ± 1.1 ^b^	555.2 ± 1.1 ^c^	32.9 ± 1.3 ^f^
*D. glomerata*	583.8 ± 1.1 ^a^	323.7 ± 1.1 ^b^	375.1 ± 1.1 ^c^	356.0 ± 1.1 ^d^	597.2 ± 1.1 ^e^	321.3 ± 1.1 ^f^
*P. arundinacea*	>800 ^a^	403.7 ± 1.1 ^c^	317.1 ± 1.2 ^e^	387.9 ± 1.1 ^d^	610.1 ± 1.1 ^b^	50.4 ± 1.2 ^f^
*L. rigidum*	>800 ^a^	683.0 ± 1.1 d	727.8 ± 1.1 ^c^	>800 ^a^	749.8 ± 1.0 ^b^	91.4 ± 2.0 ^e^
*F. rubra rubra*	>800 ^a^	529.6 ± 1.1 ^b^	623.0 ± 1.1 ^c^	485.8 ± 1.1 ^d^	559.8 ± 1.1 ^e^	28.9 ± 1.4 ^f^
*D. carota*	749.7 ± 1.1 ^b^	623.7 ± 1.1 ^c^	609.2 ± 1.1 ^d^	622.7 ± 1.2 ^c^	575.6 ± 1.1 ^e^	>800 ^a^
*M. recutita*	>800 ^a^	609.8 ± 1.1 ^c^	579.9 ± 1.1 ^d^	436.0 ± 1.2 ^f^	566.0 ± 1.2 ^e^	843.5 ± 2.1 ^b^
*T. incarnatum*	>800 ^a^	>800 ^a^	>800 ^a^	>800 ^a^	>800 ^a^	1035.0 ± 1.9 ^b^
*P. oleracea*	394.5 ± 1.1 ^a^	306.0 ± 1.1 ^c^	300.2 ± 1.1 ^d^	293.4 ± 1.1 ^e^	308.7 ± 1.0 ^b^	13.5 ± 0.15 ^f^

Letters indicate significance *p* < 0.05 between treatments (IE, F1–F4, and HBC) for each weed species, where *a* represents the higher value (two-way ANOVA and Bonferroni’s post-test).

## Data Availability

Data are contained within the article.

## Supplementary Materials

The following supporting information can be downloaded at: https://www.mdpi.com/article/10.3390/plants13192758/s1, Figure S1: Effects of the initial extract (IE), SL-enriched fractions (F1–F4), and herbicide (HBC) on the germination of *Trifolium repens*. The values are expressed as the percentage difference from the control, and Welch’s test was used for statistical analysis. Letters *a* and *b* indicate significance for *p* < 0.01 and 0.01 < *p* < 0.05, respectively; Figure S2: Effects of the initial extract (IE), SL-enriched fractions (F1–F4), and herbicide (HBC) on the germination of *Plantago lanceolata*. The values are expressed as the percentage difference from the control, and Welch’s test was used for statistical analysis. Letters *a* and *b* indicate significance for *p* < 0.01 and 0.01 < *p* < 0.05, respectively; Figure S3: Effects of the initial extract (IE), SL-enriched fractions (F1–F4), and herbicide (HBC) on the germination of *Dactylis glomerata*. The values are expressed as the percentage difference from the control, and Welch’s test was used for statistical analysis. Letters *a* and *b* indicate significance for *p* < 0.01 and 0.01 < *p* < 0.05, respectively; Figure S4: Effects of the initial extract (IE), SL-enriched fractions (F1–F4), and herbicide (HBC) on the germination of *Phalaris arundinacea*. The values are expressed as the percentage difference from the control, and Welch’s test was used for statistical analysis. Letters *a* and *b* indicate significance for *p* < 0.01 and 0.01 < *p* < 0.05, respectively; Figure S5: Effects of the initial extract (IE), SL-enriched fractions (F1–F4), and herbicide (HBC) on the germination of *Lolium rigidum*. The values are expressed as the percentage difference from the control, and Welch’s test was used for statistical analysis. Letters *a* and *b* indicate significance for *p* < 0.01 and 0.01 < *p* < 0.05, respectively; Figure S6: Effects of the initial extract (IE), SL-enriched fractions (F1–F4), and herbicide (HBC) on the germination of *Festuca rubra rubra*. The values are expressed as the percentage difference from the control, and Welch’s test was used for statistical analysis. Letters *a* and *b* indicate significance for *p* < 0.01 and 0.01 < *p* < 0.05, respectively; Figure S7: Effects of the initial extract (IE), SL-enriched fractions (F1–F4), and herbicide (HBC) on the germination of *Daucus carota*. The values are expressed as the percentage difference from the control, and Welch’s test was used for statistical analysis. Letters *a* and *b* indicate significance for *p* < 0.01 and 0.01 < *p* < 0.05, respectively; Figure S8: Effects of the initial extract (IE), SL-enriched fractions (F1–F4), and herbicide (HBC) on the germination of *Matricaria recutita*. The values are expressed as the percentage difference from the control, and Welch’s test was used for statistical analysis. Letters *a* and *b* indicate significance for *p* < 0.01 and 0.01 < *p* < 0.05, respectively; Figure S9: Effects of the initial extract (IE), SL-enriched fractions (F1–F4), and herbicide (HBC) on the germination of *Trifolium incarnatum*. The values are expressed as the percentage difference from the control, and Welch’s test was used for statistical analysis. Letters *a* and *b* indicate significance for *p* < 0.01 and 0.01 < *p* < 0.05, respectively; Figure S10: Effects of the initial extract (IE), SL-enriched fractions (F1–F4), and herbicide (HBC) on the germination of *Portulaca oleracea*. The values are expressed as the percentage difference from the control, and Welch’s test was used for statistical analysis. Letters *a* and *b* indicate significance for *p* < 0.01 and 0.01 < *p* < 0.05, respectively (Adapted from [17]).
