# Supplementary material for: Phytotoxic Activity of Sesquiterpene Lactones-Enriched Fractions from Cynara cardunculus L. Leaves on Pre-Emergent and Post-Emergent Weed Species and Putative Mode of Action
Source: Plants (Basel). 2024 Oct 1;13(19):2758. doi: 10.3390/plants13192758 (PMC11478626; doi:10.3390/plants13192758)
Supplement: Supplementary file 1 [file plants-13-02758-s001.zip › plants-3201885-supplementary.pdf]

S1 – Germination rate results for the panel of weed species

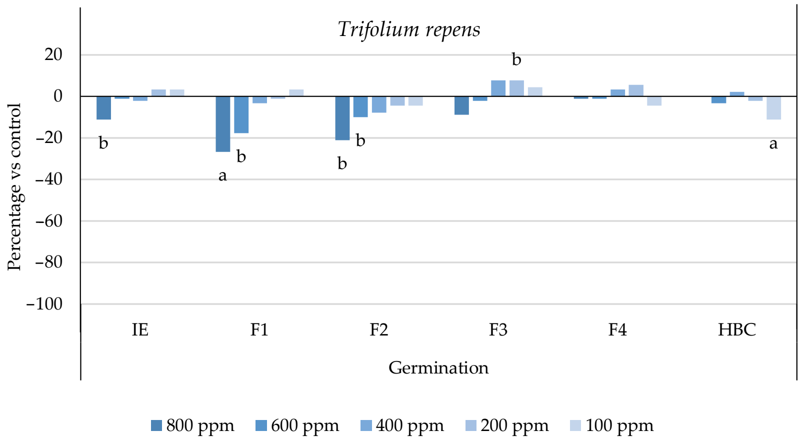

Figure S1. Effects of the initial extract (IE), SL-enriched fractions (F1–F4), and herbicide (HBC) on the germination of *Trifolium repens*. The values are expressed as the percentage difference from the control, and Welch's test was used for statistical analysis. Letters *a* and *b* indicate significance for $p < 0.01$ and $0.01 < p < 0.05$, respectively.

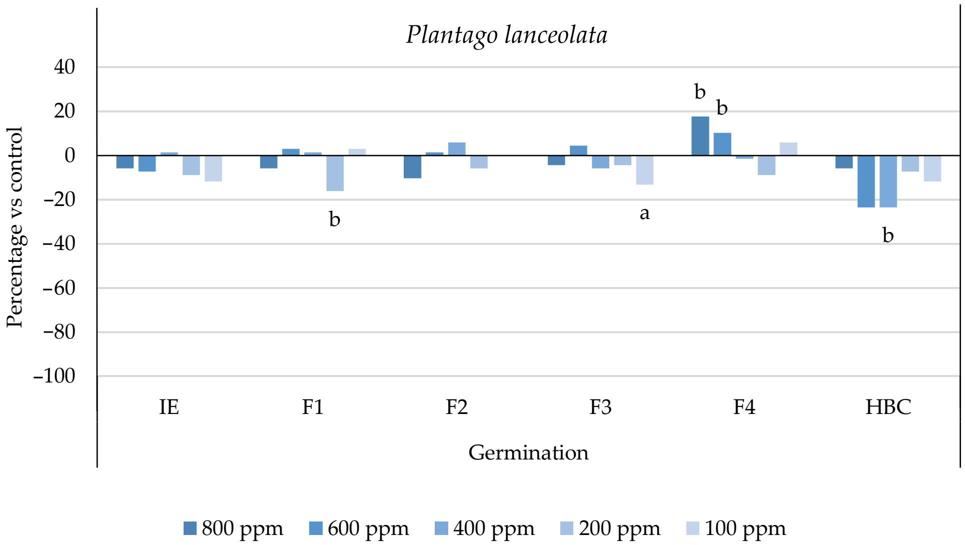

Figure S2. Effects of the initial extract (IE), SL-enriched fractions (F1–F4), and herbicide (HBC) on the germination of *Plantago lanceolata*. The values are expressed as the percentage difference from the control, and Welch's test was used for statistical analysis. Letters *a* and *b* indicate significance for $p < 0.01$ and $0.01 < p < 0.05$, respectively.

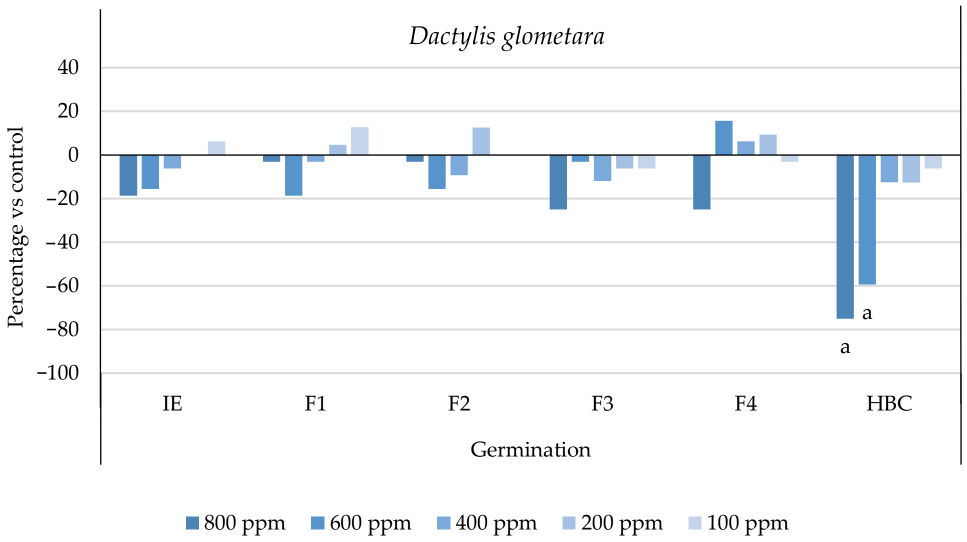

Figure S3. Effects of the initial extract (IE), SL-enriched fractions (F1–F4), and herbicide (HBC) on the germination of *Dactylis glomerata*. The values are expressed as the percentage difference from the control, and Welch's test was used for statistical analysis. Letters *a* and *b* indicate significance for $p < 0.01$ and $0.01 < p < 0.05$, respectively.

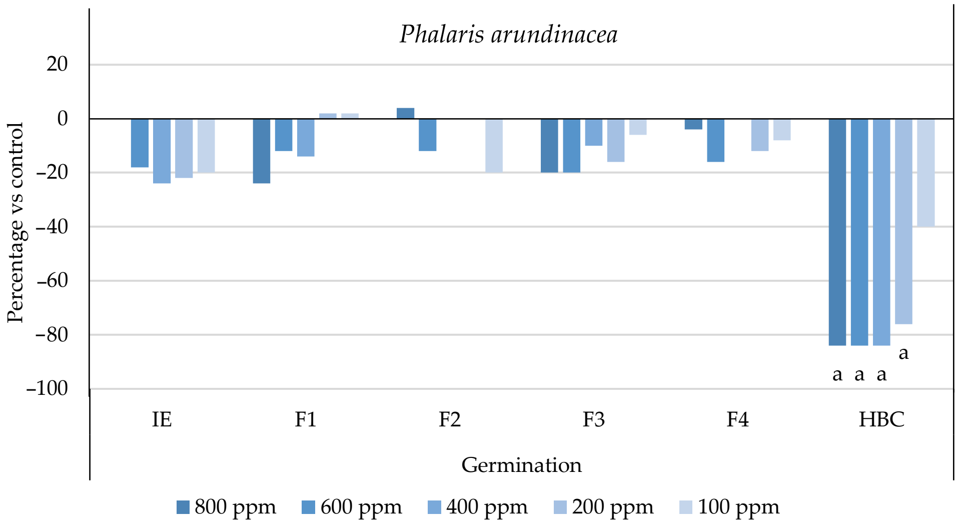

Figure S4. Effects of the initial extract (IE), SL-enriched fractions (F1–F4), and herbicide (HBC) on the germination of *Phalaris arundinacea*. The values are expressed as the percentage difference from the control, and Welch's test was used for statistical analysis. Letters *a* and *b* indicate significance for $p < 0.01$ and $0.01 < p < 0.05$, respectively.

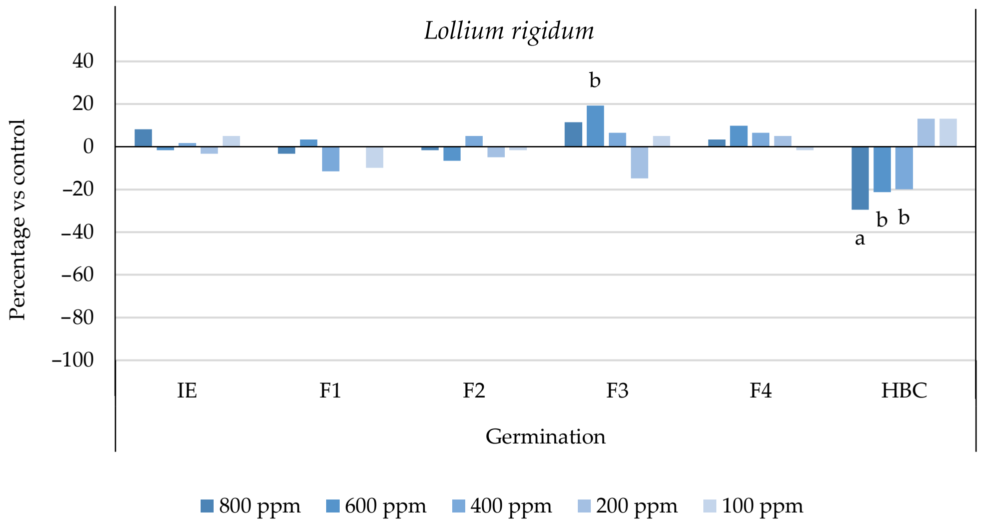

Figure S5. Effects of the initial extract (IE), SL-enriched fractions (F1–F4), and herbicide (HBC) on the germination of *Lolium rigidum*. The values are expressed as the percentage difference from the control, and Welch's test was used for statistical analysis. Letters *a* and *b* indicate significance for $p < 0.01$ and $0.01 < p < 0.05$, respectively.

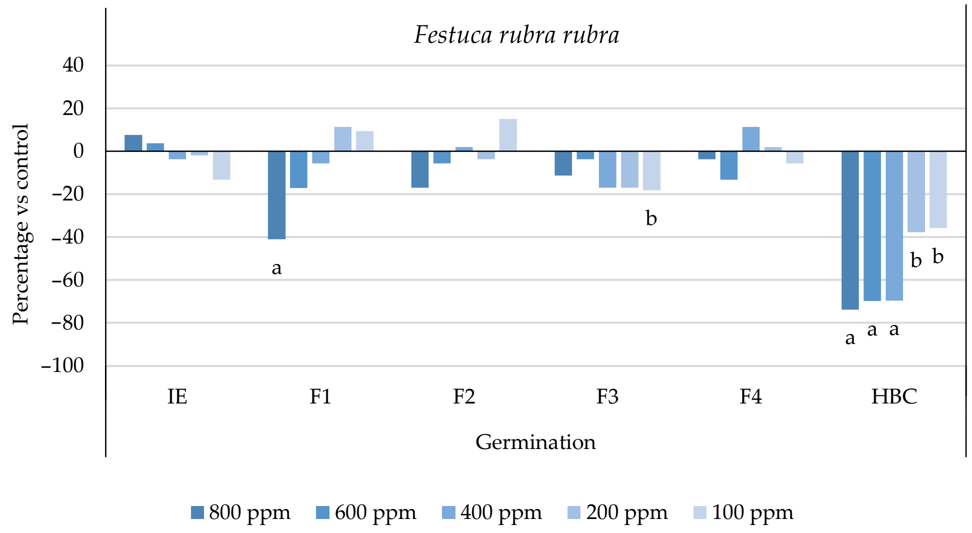

Figure S6. Effects of the initial extract (IE), SL-enriched fractions (F1–F4), and herbicide (HBC) on the germination of *Festuca rubra rubra*. The values are expressed as the percentage difference from the control, and Welch's test was used for statistical analysis. Letters *a* and *b* indicate significance for $p < 0.01$ and $0.01 < p < 0.05$, respectively.

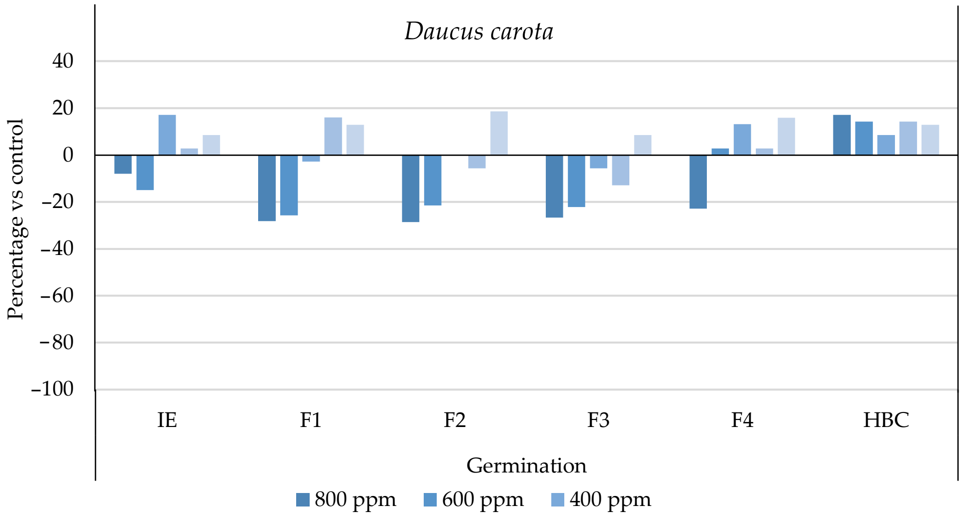

Figure S7. Effects of the initial extract (IE), SL-enriched fractions (F1–F4), and herbicide (HBC) on the germination of *Daucus carota*. The values are expressed as the percentage difference from the control, and Welch's test was used for statistical analysis. Letters *a* and *b* indicate significance for $p < 0.01$ and $0.01 < p < 0.05$, respectively.

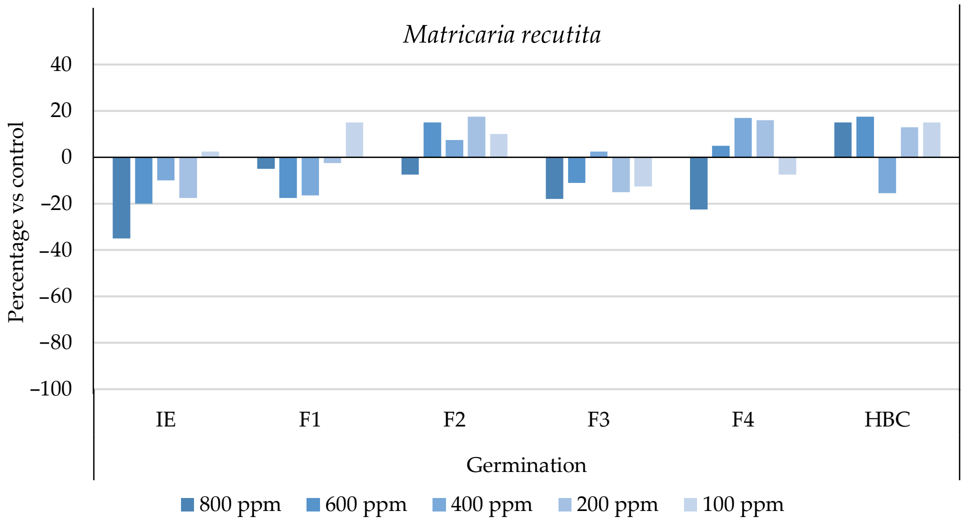

Figure S8. Effects of the initial extract (IE), SL-enriched fractions (F1–F4), and herbicide (HBC) on the germination of *Matricaria recutita*. The values are expressed as the percentage difference from the control, and Welch's test was used for statistical analysis. Letters *a* and *b* indicate significance for $p < 0.01$ and $0.01 < p < 0.05$, respectively.

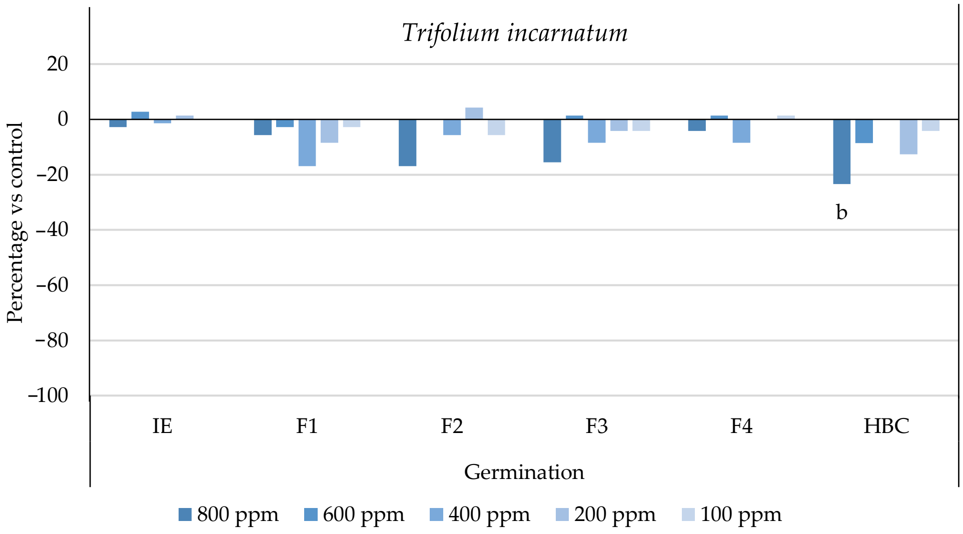

Figure S9. Effects of the initial extract (IE), SL-enriched fractions (F1–F4), and herbicide (HBC) on the germination of *Trifolium incarnatum*. The values are expressed as the percentage difference from the control, and Welch's test was used for statistical analysis. Letters *a* and *b* indicate significance for $p < 0.01$ and $0.01 < p < 0.05$, respectively.

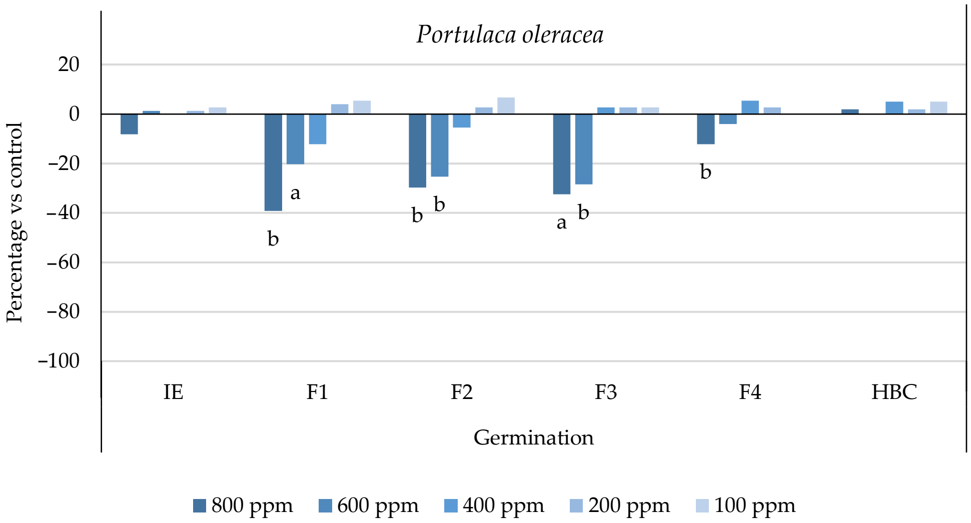

Figure S10. Effects of the initial extract (IE), SL-enriched fractions (F1–F4), and herbicide (HBC) on the germination of *Portulaca oleracea*. The values are expressed as the percentage difference from the control, and Welch's test was used for statistical analysis. Letters *a* and *b* indicate significance for $p < 0.01$ and $0.01 < p < 0.05$, respectively. (Adapted from [17]).
